# Association of job category and occupational activity with breast cancer incidence in Japanese female workers: the JACC study

**DOI:** 10.1186/s12889-020-09134-1

**Published:** 2020-07-14

**Authors:** Gita Nirmala Sari, Ehab Salah Eshak, Kokoro Shirai, Yoshihisa Fujino, Akiko Tamakoshi, Hiroyasu Iso

**Affiliations:** 1 Public Health, Department of Social Medicine, Osaka University Graduate School of Medicine, Suita-shi, Osaka, 565-0871 Japan; 2 Health Polytechnic of Jakarta III, Ministry of Health, Jakarta, Indonesia; 3 Department of Public Health, Faculty of Medicine, Minia University, El-Minia, Egypt; 4 University of Occupational and Environmental Health, Kitakyushu, Fukuoka, Japan; 5 Department of Public Health, Faculty of Medicine, Hokkaido University, Sapporo, Japan; 6 Department of Public Health Medicine, Faculty of Medicine, University of Tsukuba, Tsukuba, Japan

**Keywords:** Job, Occupational activity, Breast cancer, Incidence, Cohort study

## Abstract

**Background:**

Breast cancer represented the leading cause of cancer deaths among women in Japan. Although physical activity has been reported protective against breast cancer, scientific evidence is limited on the risk of breast cancer according to job category or occupational activity in Japanese. Our objective was to examine the association of job category and occupational activity with breast cancer incidence in Japanese female workers using the data from the Japan Collaborative Cohort (JACC) Study.

**Methods:**

A prospective cohort study involving 19,041 women aged 40–79 years who have reported their occupational data and followed-up from 1988 to 2009. All variables were assessed by a self-administered questionnaire. Cancer incidence data were obtained from 24 areas of the JACC study through cancer population data registration, or review of hospital records. The Cox proportional hazard models were operated to calculate the hazard ratios (HRs) and corresponding 95% confidence intervals (CIs).

**Results:**

There were 138 incident cases of breast cancer during 13.3 years median follow-up period. *Office* workers compared with *manual* workers were at a higher risk of breast cancer after adjusting for reproductive health factors and physical activity indicators; the multivariable HR (95% CI) was 1.65 (1.07–2.55). Also, women who had mainly a *sitting* position during work compared with those *moving* during work had the higher risk: the multivariable HR (95%CI) of 1.45 (1.01–2.12). The excess risk of breast cancer was observed for *office* workers when time spent in walking was < 30 min/ day; HR (95% CI) was 1.11 (1.01–1.23), and for women mainly at a *sitting* position during work when time spent in walking was 30–59 min or < 30 min/day; HRs (95% CIs) were 1.87 (1.07–3.27) and 1.74 (1.07–2.83), respectively.

**Conclusion:**

The job category and occupational activity were associated with risk of breast cancer incidence. A high risk was observed in *office* workers and in women with a *sitting* position during work. These observed increased risks were evident in women with less daily walking activity.

## Background

Breast cancer is the most common cancer in women worldwide. Since 1975, the incidence rate of breast cancer in Japan has been increasing, especially among women aged 40 years and above. In 2018, there were 157,000 cases of cancer mortality in Japanese women, and breast cancer was the leading cause of cancer death (9%) [1].

Prolonged exposures to estrogen critically contribute to the development of breast cancer [2–4]. Regular physical activity can reduce the adverse effect of estrogen [5]; physical activity decreases the luteal phase length of the ovulation cycle which reduces the cumulative ovarian hormone exposure [6–9].

Previous studies have indicated the association between the type of occupation and the breast cancer risk [8–12]. The initial investigations were extended to examine the effects of physical activity during [8, 10, 11] and outside work [9, 11, 12] on the risk of breast cancer. A previous report on the Japan Collaborative Cohort (JACC) study stated that the risk of mortality from breast cancer was lower among female manual workers than female office workers. However, the researchers in this report did not account for physical activity that occurs during work [13]. A study on Nordic countries reported that among the national population of women, women who work outdoors (e.g., gardeners, farmers, and woodworkers) had a lower risk of developing breast cancer compared with the entire national females [14]. Many studies on Western and Asian countries have shown that involving in physically active jobs is inversely associated with the risk of breast cancer, while sedentary or office work has a positive association [10, 15–19]. However, a pooled analysis of two case-control studies in Australia and Canada reported no association between occupational activity level and the risk of developing breast cancer [20].

The discussion thus far highlights the lack of scientific evidence for Japanese women on the risk of developing breast cancer on the basis of job category or occupational activity. Thus, we aimed in our research to assess the associations of job category *(manual, office, professional, and unclassified)* and occupational activity *(moving, mainly standing and mainly sitting)* with the risk of breast cancer incidence among Japanese female workers. We conducted stratified analyses on the basis of physical activity indicated by walking time per day (indoor, work, home and outdoor). We hypothesized that job category and occupational activity are associated with the risk of breast cancer incidence among Japanese female workers and that the level of physical activity (walking time) would influence these associations.

## Methods

### Study population and setting

A description of the population and research settings has been provided previously [21]. The JACC study is one of the largest multicenter collaborative cohort studies and was conducted from 1988 to 2009 across 45 areas in Japan, comprising three towns in Hokkaido; five towns in Tohoku district; five towns in Kanto district; one city, three towns, and two villages in Chubu district; eight towns and two villages in Kinki district; one city and one town in Chugoku district; and four cities, nine towns, and one village in Kyushu district.

For a follow-up period of approximately 20 years, data on all-cause deaths and cause-specific mortalities were assessed along with their associated risk factors. The purpose of the JACC Study was to evaluate the impact of lifestyle on human health including cancer and cardiovascular diseases.

The cancer incidence data in the JACC study were available for 24 Japanese areas with cancer incidence data registry, covering all of Japan from Hokkaido, Tohoku, Kanto, Chubu, Kinki, and Chugoku, to Kyushu. The follow-up was completed for one area each in 1994, 1999, 2000, 2002, and 2003; for two areas each in 1997, 2006, and 2008; and for 13 areas in 2009 [21].

**Fig. 1:**
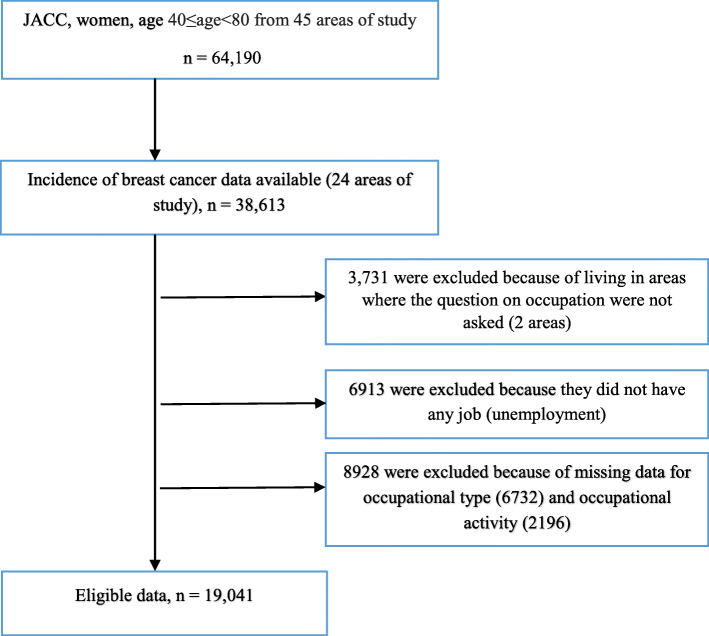
Flow chart for the calculation of respondents

Of the women living in the 24 areas, a total of 38,613 participants aged between 40 to 79 years took part in the baseline survey of the JACC study. In a self-administered questionnaire (details were published elsewhere [21]), women were asked to specify their job type into employed, self-employed, part-time, housewife, or unemployed, and accordingly we excluded 6913 women who were unemployed. We further excluded 3731 women living in two areas where the occupation-related questions were not asked, and 8928 women who did not answer the occupation-related questions (6732 women with missing data on the job category and 2196 women with missing data on the occupational activity). Thus, the total number of eligible respondents for this study was 19,041 women (Fig. 1).

### Assessments of job category, occupational activity and other covariates

In a self-administered questionnaire, participants were further asked to classify their job category into *office*, *manual* and *others*. Based on the Japan Standard Industry Classification (JSIC) published by the Ministry of Internal Affairs and Communication of Japan which included a wide range of specific jobs coded from 1 to 99 [22], the participants who answered the job category as *others* were allocated to *manual* (for examples; sales, restaurant, forestry, fisheries workers, etc.), *office* (for examples; clerk, postal, management staff, etc.), *professional* (for examples; health worker, judges, accountant, musician, etc.) and *unclassified job* categories. For the current research, we reclassified job categories into four categories namely *manual, office*, *professional* and *unclassified*. The occupational activity was classified according to the position during work as *moving*, *mainly standing* and *mainly sitting*.

Other covariates included on the questionnaire were used as confounding factors to strengthen the results: body mass index (BMI) calculated as weight in kg divided by squared height in m (< 18.5, 18.5–24.9, 25–29.9, ≥ 30 kg/m^2^); smoking status (never, ex-smoker, or current smoker); alcohol intake (never, ex-drinker, or current drinker); education (≤ 15 years, or ≥ 16 years), family history of cancer (yes or no), feeling daily stress (stressful, normal, or less stressful); and reproductive health factors, such as marital status (married and unmarried), age at menarche (< 14 years or ≥ 14 years), age at menopause (≤ 50 years or > 50 years), and number of deliveries (0, 1, 2, or ≥ 3). The last group of confounding factors were those specific to our priori set hypothesis for physical activity indicators: walking time (< 30 min/ day, 30–59 min/ day, or ≥ 1 h/ day) and sport time (< 1 h/ week, 1–2 h/ week, or ≥ 3 h/ week). Dummy variables representing missing observations of each confounder were added to the model.

### Outcome assessment

Breast cancer incidence data, comprising the diagnosis date and primary site of cancer, were confirmed through records in the population-based cancer registries for each cohort study area, supplemented by a systematic review of death certificates and medical records from major local hospitals in the 24 study areas [21, 23–25]. Breast cancer diagnosis was determined as per code C50 in the International Classification of Diseases, ninth revision (ICD-9), used from the baseline JACC survey to 1994; and the tenth revision (ICD-10) used post-1995 [21, 23–27]; in both, C50 codes for malignant neoplasm of the breast, not including ductal carcinoma, in situ.

### Statistical analysis

The baseline risk characteristics were presented as mean values (standard deviations) and proportions. The risk of breast cancer according to the job category and occupational activity was assessed by estimating the hazard ratios (HRs) with 95% confidence intervals (CIs) using the Cox proportional hazards regression models adjusted for the abovementioned confounding factors.

Additionally, the associations of the breast cancer risk with the job category and occupational activity were tested after the stratification by walking time (< 30 min/day, 30–59 min/day, or ≥ 1 h/day).

Next, a sensitivity analysis was conducted by excluding participants who reported they were housewives under the job type question. The objective of this analysis was to determine the strength of the relationship that job category and occupational activity has with the incidence of breast cancer.

### Ethical statement

Informed consent was obtained from the study participants or their community representatives prior to their participation in this study. The ethics committees of Nagoya University School of Medicine and Osaka University approved the protocol for this study.

## Results

### Participants characteristics

Table 1 shows the baseline characteristics of Japanese women according to the job category and occupational activity. The averages age and BMI of women were between 50.1 to 59.2 years and 22.4 to 22.8 kg/m^2^, respectively. Women who worked in *office* and women engaged in *moving* occupational activity were more likely to be drinkers. A family history of breast cancer was more commonly observed among women who were *manual* workers and women reporting *mainly a standing* position during work. Women who reported working in *office* jobs and women in *mainly a sitting* position during work had high perceived stress levels, were less likely to be multigravida for ≥3 children and were less likely to walk for ≥1 h/day.

**Table 1:** Women’s baseline characteristics according to job category and occupational activity

Parameters	Job category				Occupational activity		
	Manual	Office	Professional	Unclassified	Moving	Standing	Sitting
Number at risk	11,464	2388	3727	1462	9453	1033	5570
Number of breast cancer	85	31	18	4	60	4	63
Age, mean, mean (SD)	53.3 (8.5)	50.1 (7,7)	57.4 (8.7)	59.2 (9.9)	54.6 (8.6)	53.2 (8.5)	51 (8.1)
Body mass index, mean (SD)	22.8 (2.9)	22.5 (2.8)	22.7 (2.9)	22.4 (2.9)	22.8 (2.9)	23.1 (3.1)	22.7 (2.9)
Smoking (%)							
Never smoker	87.9	87.6	85.8	89.7	86.4	81.8	88.7
Former smoker	1.0	1.1	1.1	1.5	1.1	1.7	1.0
Current smoker	5.0	6.2	10.9	3.8	4.8	7.1	4.8
Alcohol intake (%)							
Never drinker	70.1	63.1	69.7	76.1	68.8	66.1	67.6
Former drinker	1.2	1.4	1.2	6.7	1.4	23.2	1.1
Current drinker	16.7	21.7	15.2	7.2	18.5	17.3	17.2
Education ≤15 year (%)	33.7	11.7	29.0	40.2	33.1	33.1	22.3
Family history of breast cancer (%)	8.4	7.5	1.7	65.7	8.8	10.6	5.4
Feeling daily stress (%)							
Stressful	21.3	33.6	19.7	11.0	22.2	24.6	28.2
Normal	56.4	48.6	61.3	29.6	60.8	61.2	56.4
Less stressful	13.3	11.2	15.3	6.5	14.0	12.1	13.5
Currently married (%)	92.8	91.1	91.1	51.9	94.4	93.2	93.2
Menarche age, mean (SD)	14.7 (1.8)	14.1 (1.6)	14.8 (1.7)	15.4 (1.8)	14.7 (1.7)	14.6 (1.8)	14.4 (1.7)
Menopause age, mean (SD)	48.5 (4.6)	48.4 (5.1)	48.7 (4.4)	48.8 (4.5)	48.4 (4.8)	48.1 (4.6)	48.7 (4.3)
Number of delivery (%)							
0	4.0	5.2	3.2	8.3	3.6	4.4	3.8
1	6.3	8.2	6.3	5.7	6.0	7.7	7.7
2	39.3	45.1	32.7	29.3	36.1	39.6	44.9
≥ 3	46.0	35.4	51.5	52.1	49.8	42.9	37.4
Walking time (/day)							
Never or < 30 min	26.8	35.8	16.3	16.7	20.0	33.1	34.5
30–59 min	18.3	23.8	16.7	11.3	17.5	21.6	22.1
≥ 1 h	50.2	34.6	59.3	26.3	58.0	41.5	40.9
Sport time (/week)							
< 1 h	74.6	70.4	70.6	39.7	74.0	79.9	74.9
1–2 h	12.9	17.5	11.1	7.2	12.3	12.5	15.4
≥ 3 h	7.5	7.1	10.0	6.4	8.9	4.5	7.0

The median follow-up time for this research was 13.3 years, and the number of newly diagnosed breast cancer cases was 138 among 19,041 women at risk.

### Job category and risk of breast cancer

Table 2 shows that women who worked in an *office* were at a higher risk of developing breast cancer than *manual* workers even after controlling for reproductive health factors, education and BMI. However, the risk was slightly attenuated after controlling for the physical activity indicators; the multivariable HR (95% CI) was 1.65 (1.07–2.55) in the fully adjusted model. There was no excess risk of breast cancer in *professional* or *unclassified* workers compared with *manual* workers.

**Table 2:** Age-adjusted and multivariable hazard ratios (95% confidence intervals) of incident breast cancer according to job category and occupational activity

	Number at risk	Number of breast cancer	Person-years	Age-adjusted HR (95%CI)	Multivariable HR (95% CI)	
					Model 1^a^	Model 2^b^
Job category						
1. Manual	11,464	85	157,490	1.00 (reference)	1.00 (reference)	1.00 (reference)
2. Office	2388	31	33,150	1.70 (1.12–2.58)	1.68 (1.10–2.59)	1.65 (1.07–2.55)
3. Professional	3727	18	49,292	0.70 (0.42–1.18)	0.72 (0.42–1.23)	0.80 (0.47–1.36)
4. Unclassified	1462	4	15,510	0.49 (0.17–1.35)	0.72 (0.17–2.99)	0.38 (0.05–2.80)
Occupational activity						
1. Moving	9453	60	123,944	1.00 (reference)	1.00 (reference)	1.00 (reference)
2. Standing	1033	4	13,364	0.61 (0.22–1.68)	0.47 (0.14–1.52)	0.46 (0.14–1.49)
3. Sitting	5570	63	82,071	1.57 (1.09–2.25)	1.51 (1.04–2.19)	1.45 (1.01–2.12)

^a^ Adjusted further for BMI, smoking status, alcohol intake, education, family history of cancer, feeling daily stress, marital status, age of menarche, age of menopause and number of delivery

^b^Adjusted further for walking time and sport time

### Occupational activity and risk of breast cancer

A higher risk of breast cancer was observed among women with *mainly a sitting* occupation activity than in those with a *moving* occupation activity. The association was slightly attenuated after controlling for the physical activity indicators; the multivariable HR (95% CI) was 1.45 (1.01–2.12) (Table 2).

### Sensitivity analysis

Excluding the 3626 women who reported their type of job as housewives attenuated the association of *office* work and *mainly a sitting* occupational activity with breast cancer incidence risk. The multivariable HRs (95% CIs) were 1.54 (0.97–2.44) and 1.45 (0.97–2.18), respectively (Additional file 1).

### Stratified analyses

Table 3 shows the HRs of breast cancer incidence for each job category and occupational activity after stratification by the daily walking time. The observed associations of *office* versus *manual* job categories and *sitting* versus *moving* occupational activities with the risk of higher breast cancer were evident for women who walked less frequently on a daily basis. The multivariable HR (95% CI) for breast cancer among *office* versus *manual* workers who walked for < 30 min/ day was 1.11 (1.01–1.23). The respective risk estimates for women with a *sitting* versus a *moving* occupational activity were 1.87 (1.07–3.27) among women who walked 30–59 min/ day and 1.74 (1.07–2.83) among those who walked < 30 min/ day.

**Table 3:** Age-adjusted and multivariable hazard ratios (95% confidence intervals) of incident breast cancer according job category and occupational activity after stratification by daily walking time

		Daily walking time		
		≥ 1 h	30–59 min	< 30 min
Occupation				
*1. Manual*	Number at risk	5755	2099	3071
	Number of breast cancer	38	23	23
	Person-years	80,397	28,710	41,034
	Age-adjusted HR (95%CI)	1.00 (reference)	1.00 (reference)	1.00 (reference)
	Multivariable OR (95%CI)^a^	1.00 (reference)	1.00 (reference)	1.00 (reference)
*2. Office*	Number at risk	826	568	855
	Number of breast cancer	11	8	12
	Person-years	11,861	7895	11,626
	Age-adjusted HR (95%CI)	1.15 (0.98–1.34)	1.14 (0.98–1.31)	1.17 (1.01–1.23)
	Multivariable OR (95%CI)^a^	1.12 (0.95–1.32)	1.12 (0.97–1.30)	1.11 (1.01–1.23)
*3. Professional*	Number at risk	2211	622	609
	Number of breast cancer	8	4	6
	Person-years	29,219	8127	7907
	Age-adjusted HR (95%CI)	0.92 (0.82–1.02)	0.99 (0.88–1.13)	1.04 (0.95–1.14)
	Multivariable OR (95%CI)^a^	0.92 (0.83–1.02)	0.96 (0.83–1.12)	1.04 (0.95–1.14)
*4. Unclassified*	Number at risk	384	165	244
	Number of breast cancer	1	0	0
	Person-years	4031	1532	2264
	Age-adjusted HR (95%CI)	0.93 (0.76–1.13)	NA	NA
	Multivariable OR (95%CI)^a^	0.96 (0.79–1.17)	NA	NA
p for interaction				0.03
Occupation activity				
*1. Moving*	Number at risk	3768	1215	1956
	Number of breast cancer	26	11	11
	Person-years	52,811	16,989	26,395
	Age-adjusted HR (95%CI)	1.00 (reference)	1.00 (reference)	1.00 (reference)
	Multivariable OR (95%CI)^a^	1.00 (reference)	1.00 (reference)	1.00 (reference)
*2. Standing*	Number at risk	729	520	513
	Number of breast cancer	7	7	795
	Person-years	10,333	7138	12
	Age-adjusted HR (95%CI)	NA	0.80 (0.11–5.77)	1.56 (0.49–4.98)
	Multivariable OR (95%CI)^a^	NA	NA	1.56 (0.49–4.99)
*3. Sitting*	Number at risk	4679	1719	2028
	Number of breast cancer	25	17	18
	Person-years	62,365	22,138	25,777
	Age-adjusted HR (95%CI)	1.51 (0.93–2.43)	2.12 (1.24–3.63)	1.84 (1.14–2.97)
	Multivariable OR (95%CI)^a^	1.38 (0.85–2.27)	1.87 (1.07–3.27)	1.74 (1.07–2.83)
p for interaction				0.006

^a^Adjusted further BMI, smoking status, alcohol intake, education, family history of cancer, feeling daily stress, marital status, age of menarche, age of menopause and number of delivery, and sport time

## Discussion

This cohort study supports the evidence for the associations of job category and occupational activity with the risk of breast cancer incidence among Japanese women. *Office* workers were at a higher risk of developing breast cancer than *manual* workers. In addition, women who were *mainly in a sitting* position during work were at a higher risk of developing breast cancer than those who were *moving* during work. These associations were evident for women who report less walking activity on a daily basis.

Several previous studies on non-Asian [14, 28, 29] and Asian population [7, 13, 16, 30–32] have shown similar associations between occupation and risk of breast cancer. A case-control study in Massachusetts on women aged ≤74 years (6835 cases of breast cancer and 9453 controls) reported a higher risk of breast cancer among women who worked in administrative occupations (multivariable OR = 1.15, 95% CI = 1.06–1.24) than in housewives [28]. Another study highlighted that among 7.5 million Nordic women, women with manual jobs reported a lower risk of breast cancer, such as gardeners (age-adjusted relative risk: RR = 0.76, 95% CI = 0.74–0.78), farmers (RR = 0.80, 95% CI = 0.78–0.82), and woodworkers (RR = 0.75, 95% CI = 0.70–0.81), whereas the risk was higher among women who were dentists (RR = 1.43, 95% CI = 1.31–1.56) and physicians (RR = 1.35, 95% CI = 1.26–1.46) in reference to the entire national female study population [14]. In a study on women in Shanghai, the lowest standardized incidence ratios (SIR) for breast cancer were observed for *manual* workers, such as women working in construction (SIR = 0.5, 95% CI = 0.3–0.9) or production (SIR = 0.6, 95% CI = 0.5–0.8), whereas the highest SIRs were found in women who worked in *office* such as researchers (SIR = 3.3,95% CI = 1.4–6.5) and administrative clerks (SIR = 1.6,95% = 1.3–1.9) [31]. Similar results were observed in another Chinese study, where an inactive job classification was associated with higher SIRs not only of breast cancer, but also for uterine and ovarian cancer [7]. A previous report on the JACC study suggested that, compared with *office* workers, female *manual* workers were at a lower risk of breast cancer mortality (area-adjusted HR = 0.41, 95% CI = 0.19–0.92) [13].

In this study, female workers were at a higher risk of developing breast cancer if their occupational activity involved mainly a *sitting* rather than a *moving* position during work. The association between occupational activity and risk of breast cancer was consistent with the findings from previous non-Asian [8, 18, 19, 33–36] and Asian [7, 13, 16, 24, 30, 31, 37] studies. A case-control study of women aged 35–93 years in Stettin, Poland found that women aged > 55 years with a medium-level occupational physical activity were at a lower risk of developing breast cancer than those who had a sedentary occupational activity (multivariable OR = 0.40, 95% CI 0.20–0.81) [35]. Similar results were also reported by a Swedish population-based cohort study on 29,524 women (with 1506 breast cancer cases within the 24 years of follow-up). Compared with women who worked in non-sedentary occupations, the risk of developing breast cancer was higher for women who worked in sedentary occupations (HR = 1.54; 95% CI = 1.20–1.96), but not for women who worked in mixed occupations (HR = 1.08; 95% CI = 0.85–1.37) [19]. A prospective cohort study in China on a population of 73,049 Chinese women also reported that women with the lowest average occupational sitting time (< 3.69 h/ day) were at a lower risk of developing breast cancer (HR = 0.69; 95% CI 0.57–0.54) than those with an average sitting time ≥ 4 h/ day [16]. Two case-control studies in other Asian countries (India and China) have also reported that high occupational activity and exercise can help reduce the risk of breast cancer [37, 38].

The results of this study are also in line with the conclusion of two meta-analyses. The first meta-analysis was conducted on 14 case-control studies and 7 cohort studies with 2,625,772 participants and 82,630 breast cancer patients, and found an increased risk of breast cancer in women with a sedentary behaviour, including prolonged occupational sitting time (OR = 1.10, 95% CI = 1.02–1.18) [39]. The second meta-analysis was performed on 31 prospective studies involving 63,786 women and examined the association between physical activity and breast cancer risk [15]. This meta-analysis found that physical activity from occupational and non-occupational settings (i.e., leisure-time and household activities) was inversely associated with the risk of breast cancer. The analysis reported that the multivariable RR with 95% CI was 0.87 (0.83–0.91) for non-occupational activity and 0.90 (0.83–0.97) for occupational activity.

On the other hand, a prospective cohort study of 9539 twin Swedish women aged 42–70 years indicated no association of total physical activity, including both leisure and work activities with the risk of breast cancer; the age-adjusted RR (95% CI) was 0.9 (0.7–1.2) for active occupations in reference to sedentary occupations [40]. In addition, the prospective cohort study by European Prospective Investigation into Cancer and Nutrition (EPIC) found no difference in the risk of breast cancer among sedentary, standing and manual and heavy workers. In comparison with sedentary activities, the multivariable-adjusted HRs (95%CIs) were 0.92 (0.81–1.05) for standing, 1.08 (0.91–1.29) for manual and heavy manual labour activities [41]. Our study did not examine the risk of breast cancer incidence for the occupational activity of *mainly standing* position during work because of the small number of cases; however, a previous report on the JACC study stated that women who were generally required to stand during work were at a higher risk of mortality due to breast cancer (HR = 3.00, 95% CI = 1.06–8.42) than women who did sedentary work only [13]. The variations in the results between the EPIC study and our analysis can be attributed to the different definitions of the occupational activity categories and assessments of the physical activity. For example, the EPIC study [41], accounted for unemployed women under sedentary occupational activities (reference group), whereas our study excludes unemployed women. Previous studies have shown that unemployed women are at a higher risk of breast cancer [42]. Moreover, it is well-known that unemployment hinders breast cancer screening behavior among females in both Japan [43] and the United States [44].

In this study, the excess risk of breast cancer was still evident for *office* workers who walked for a duration of < 30 min/ day, when compared to *manual* workers. Also, an excess risk of breast cancer was observed among women who *mainly sat* during work compared to those who *moved* during work, and was particularly high when their walking duration was < 1 h/ day. This supports the evidence on the effect of physical activity, not only occupational, but also leisure activities, on the risk of developing breast cancer [6, 7, 10, 12, 15, 24]. A Swedish case-control study that included 3455 controls and 3347 post-menopausal cases of breast cancer found that working women with sedentary jobs and who rarely engaged in leisure-time activity were at a three-fold higher risk of breast cancer than women who were active within both inside and outside of workplace [6]. A previous report on the JACC study showed that the multivariable HR (95% CI) for breast cancer incidence was 0.45 (0.25–0.78) among women who walked ≥1 h per day and engaged in sport for ≥1 h/ day compared with women who walked < 1 h/ day and engaged in sport for < 1 h/ day [24]. However, the report did not account for physical activity during work or the job category. The Shanghai Women’s Health Study examined a joint effect of occupational sitting and adulthood exercise on the risk of breast cancer in post-menopausal women and showed a 30% risk reduction of breast cancer incidence in women who had either less occupational sitting time (≤ 2.1 h/ day) or who engaged in adequate exercise (≥ 8 metabolic equivalent units/ week) compared with women who reported both longer occupational sitting time (≥ 4 h/ day) and inadequate exercise (< 8 metabolic equivalent units/week). However, contrary to the present study, that study found no statistically significant interaction between occupational and leisure activities [16].

The breast cancer incidence risk is closely related to imbalances in sex hormones, and this is one mechanism that could explain our findings. High exposure to estrogen and other ovarian hormones plays an important role in the development of breast cancer [5, 6, 45]. Hormone imbalance is closely related to lifestyle factors, such as being physically inactive (indoors and outdoors) [2, 45]. Physical activity reduces the level of steroid sex hormones and this reduction decreases the risk of hormone-related cancers [45–47]. The 17-β-estradiol (E2) is an indicator of the development and prognosis of breast cancer [5]. A Polish study on urban and rural women showed that the concentration of this estradiol in saliva was 21% higher in low-activity groups than in high-activity groups [5]. Reduced exposures to insulin and insulin-like growth factors (IGFs) are potent stimulators of cell growth related to the development of breast cancer [39–41]. Physical activity increases the production of insulin-like growth factor-binding protein-1 (IGFBP-1), which down regulates IGFs [48, 49].

Our study makes several important contributions. We investigated a large population-based sample of women, with a high response rate and a long follow-up period. In addition, the prospective cohort design of our research allowed us to reduce several types of bias, especially recall bias. However, several limitations of this study need to be addressed. The use of a simple questionnaire at baseline to collect information about the job category, activity during work, and physical activity in general could result in some inevitable misclassifications. In addition, while the main analyses of job category and occupational activity with the risk of breast cancer beard reasonable numbers of cases in each category, the stratification analyses had small numbers of cases for certain categories and thus, lacked sufficient power to detect real associations. Therefore, the results from our stratified analyses should be carefully interpreted. The occupational and covariates data were obtained once and were self-reported; such data could have been changed during the extensive follow-up period. Further, some women reported being a housewife while classified their job category as *office* (*n* = 354), *manual* (*n* = 2265), or *professional* (*n* = 688); this might have led to some misclassifications by women in such job categories and still taking care of the housework. Excluding these participants attenuated the association, although the trend for a positive association of the *office* jobs and *sitting* occupational activity with the risk of breast cancer persisted. Finally, while we controlled for a wide range of possible confounders, the effect of certain residual confounding factors, such as the use of hormone replacement therapy, remains to be addressed.

## Conclusion

In conclusion, the job category and occupational activity were associated with breast cancer incidence risk. Women who worked in *office* and those whose jobs mainly required them to *sit* were at the higher risk of developing breast cancer. The higher risk of breast cancer in *office* jobs and *mostly sitting* during work was evident among women whose walking activities were limited. Our findings imply that women who work in *offices* and *mainly sit* during the workday should increase their physical activity to reduce their risk of developing breast cancer.

## Supplementary information

**Additional file 1: Supplementary Table**. Age-adjusted and multivariable hazard ratios (95% confidence intervals) of incident breast cancer according to job category and occupational activity after exclusion of housewives in occupation

## Data Availability

The data can be made available from the corresponding author under reasonable request.

## Abbreviations

JACC: Japan Collaborative Cohort
EPIC: European Prospective Investigation into Cancer and Nutrition
