# Supplementary material for: Association of job category and occupational activity with breast cancer incidence in Japanese female workers: the JACC study
Source: BMC Public Health. 2020 Jul 14;20:1106. doi: 10.1186/s12889-020-09134-1 (PMC7362447; doi:10.1186/s12889-020-09134-1)
Supplement: Supplementary file 1 — Additional file 1: Supplementary Table. Age-adjusted and multivariable hazard ratios (95% confidence intervals) of incident breast cancer according to job category and occupational activity after exclusion of housewives in occupation [file 12889_2020_9134_MOESM1_ESM.docx]

| **Additional file 1.**  **Supplement Table. Age-adjusted and multivariable hazard ratios (95% confidence intervals) of incident breast cancer according to job category and occupational activity after exclusion of housewives in occupation** | | | | | | |  |
| --- | --- | --- | --- | --- | --- | --- | --- |
|  |  |  |  |  |  |  |  |
|  | Number at risk | Number of breast cancer | Person-years | Age-adjusted HR (95%CI) | Multivariable HR (95% CI) | | |
|  |  |  |  |  | Model 1 * | Model 2** | |
| Job category |  |  |  |  |  |  | |
| *1. Manual* | 8761 | 69 | 121,456 | 1.00 (reference) | 1.00 (reference) | 1.00 (reference) | |
| *2. Office* | 2329 | 28 | 32,407 | 1.50 (0.96 - 2.34) | 1.53 (0.98 - 2.41) | 1.54 (0.97 - 2.44) | |
| *3. Professional* | 3100 | 14 | 40,350 | 0.63 (0.35 – 1.13) | 0.61 (0.33 - 1.18) | 0.67 (0.36 - 1.24) | |
| *4. Unclassified* | 1225 | 4 | 13,091 | 0.55 (0.20 – 1.54) | 1.01 (0.24 – 4.24) | 0.54 (0.07 – 4.01) | |
|  |  |  |  |  |  |  | |
|  |  |  |  |  |  |  | |
| Occupational activity |  |  |  |  |  |  | |
| *1. Moving* | 7574 | 51 | 99,210 | 1.00 (reference) | 1.00 (reference) | 1.00 (reference) | |
| *2. Mainly standing* | 876 | 3 | 11,285 | 0.51 (0.16 - 1.64) | 0.35 (0.08 - 1.46) | 0.35 (0.08 - 1.47) | |
| *3. Mainly sitting* | 4797 | 56 | 71,780 | 1.50 (1.02 - 2.21) | 1.47 (0.99 - 2.19) | 1.45 (0.97 - 2.18) | |
| *Adjusted further for BMI, smoking status, alcohol intake, education, family history of cancer, feeling daily stress, marital status, age of menarche, age of menopause and number of delivery. | | | | | | |  |
| **Adjusted further for walking time and sport time | | | | | | |  |
|  |  |  |  |  |  |  | |
|  |  |  |  |  |  |  | |
|  |  |  |  |  |  |  | |
